# Upper Tract Urothelial Carcinoma (UTUC) Diagnosis and Risk Stratification: A Comprehensive Review

**DOI:** 10.3390/cancers15204987

**Published:** 2023-10-14

**Authors:** Masoud Bitaraf, Mahmood Ghafoori Yazdi, Erfan Amini

**Affiliations:** Uro-Oncology Research Center, Tehran University of Medical Sciences, Tehran 1419733141, Iran; sm-bitaraf@alumnus.tums.ac.ir (M.B.); ghafoori5@gmail.com (M.G.Y.)

**Keywords:** upper tract urothelial carcinoma, UTUC, diagnosis, risk stratification, nomogram, prognosis

## Abstract

**Simple Summary:**

To choose the appropriate treatment for patients with upper tract urothelial carcinoma (UTUC), proper diagnosis and risk assessment of the disease is mandatory. This study reviews some of the diagnostic tools, and the patient- and disease-related prognostic factors that affect the outcome. Predictive tools designed by these factors help determine which patients should undergo radical nephroureterectomy. Other tools help post-operative decisions regarding the use of chemotherapy and planning follow-up sessions. The available pre-operative predictive tools and post-operative nomograms are discussed. A revision of the current classification of patients to low- and high-risk groups is recommended, to expand the number of patients benefiting from kidney-sparing surgeries.

**Abstract:**

Diagnosis and risk stratification are cornerstones of therapeutic decisions in the management of patients with upper tract urothelial carcinoma (UTUC). Diagnostic modalities provide data that can be integrated, to provide nomograms and stratification tools to predict survival and adverse outcomes. This study reviews cytology, ureterorenoscopy and the novel tools and techniques used with it (including photodynamic diagnosis, narrow-band imaging, optical coherence tomography, and confocal laser endomicroscopy), and biopsy. Imaging modalities and novel biomarkers are discussed in another article. Patient- and tumor-related prognostic factors, their association with survival indices, and their roles in different scores and predictive tools are discussed. Patient-related factors include age, sex, ethnicity, tobacco consumption, surgical delay, sarcopenia, nutritional status, and several blood-based markers. Tumor-related prognosticators comprise stage, grade, presentation, location, multifocality, size, lymphovascular invasion, surgical margins, lymph node status, mutational landscape, architecture, histologic variants, and tumor-stroma ratio. The accuracy and validation of pre-operative predictive tools, which incorporate various prognosticators to predict the risk of muscle-invasive or non-organ confined disease, and help to decide on the surgery type (radical nephroureterectomy, or kidney-sparing procedures) are also investigated. Post-operative nomograms, which help decide on adjuvant chemotherapy and plan follow-up are explored. Finally, a revision of the current stratification of UTUC patients is endorsed.

## 1. Introduction

Upper tract urothelial carcinoma (UTUC) is a rare and heterogeneous disease that accounts for up to 5% of all urothelial neoplasms [1]. Accurate diagnosis and risk stratification are indispensable for determining the optimal therapeutic management for each individual patient. While the standard therapy for UTUC used to be radical nephroureterectomy (RNU), kidney-sparing surgeries (KSS) have emerged as an alternative and are increasingly being utilized. KSS includes endoscopic management, such as ureteroscopy or a percutaneous approach, as well as segmental ureterectomy that preserves the ipsilateral renal unit [2].

Diagnostic tools guide stratification, which in turn, leads to decisions regarding type of surgery, chemotherapy, and follow-up strategy. However, UTUC diagnosis and risk stratification can be challenging, and various prognostic models, nomograms, and new diagnostic tools have been developed to guide risk stratification and improve UTUC diagnosis accuracy. This review aims to provide a comprehensive overview of the current state of UTUC diagnosis and risk stratification, including the use of cytology, endoscopic evaluation, prognostic factors, and nomograms. Imaging modalities and novel biomarkers are discussed in another article from the same issue. Pre- and post-operative predictive tools are discussed and the need for novel classifications is highlighted.

## 2. Methods

This is a narrative review. Medline was searched through Pubmed from commencement to 22 April 2023. Studies on diagnosis and risk stratification of UTUC were included only after assessment of methodological rigor, and conceptual consistency. No language or article type limit was applied. Data on imaging and novel biomarkers were excluded.

## 3. Diagnostic Tests

### 3.1. Urine Cytology

Once carcinomas of the bladder and prostatic urethra are ruled out, abnormal cytology may point to high-grade UTUC. Voided urinary cytology has a sensitivity of 11% to 71.1%. Performing selective cytology, or combining cytology with biopsy improves its detection rates [3]. Selective urinary cytology is the process of obtaining urine samples from ureters separately. It is highly sensitive to high-grade tumors, including carcinoma in situ (CIS) [4]. Zhao et al. suggested that biopsy or cytology alone yields a sensitivity of about 60% for high-grade UTUC. While combining the two, increases the sensitivity to 85% [3]. Barbotage cytology is the process of infusing saline into the urinary tract, using a flexible ureteroscope and gently flushing the fluid in and out, to obtain mucosal cells. Barbotage cytology is accurate in diagnosing UTUC with a detection rate of up to 91% [5]. Overall, urine cytology is an available, cost-effective and simple test that has retained its application in UTUC diagnosis despite its limitations.

### 3.2. Ureterorenoscopy and Biopsy

Formerly, UTUC diagnosis was mainly based on imaging. Emerging new kidney-sparing and neo-adjuvant treatments, highlighted the importance of ureterorenoscopy (URS). It is now an integral part of UTUC workup, which helps determine the size, location, and architecture of the suspicious lesions, and obtain biopsy samples [6]. However, concerns remain regarding diagnostic URS’s impact on oncological outcomes. A meta-analysis on 5489 patients indicated that URS plus biopsy is associated with worse intravesical recurrence-free survival (IVRFS) following RNU (Hazard ratio (HR): 1.44, 95% Confidence interval (CI): 1.29–1.61, *p* < 0.001), but it does not affect long-term survival outcomes. Diagnostic URS without biopsy was not associated with worse IVRFS [7]. Table 1 summarizes the studies assessing the association between URS, IVR (intravesical recurrence), and RFS (recurrence-free survival) [8,9,10,11,12,13,14,15,16,17,18,19,20,21,22]. A study on 143 patients demonstrated a pathological phenotype-specific association between pre-operation URS and oncological outcomes, as the subgroup of patients with non-papillary and ≥pT3 UTUC had poorer overall and progression-free survival [23].

Due to limitations of white light URS, novel tools are being experimented with the aim of improving the detection rate and increasing sensitivity and specificity. Photodynamic diagnosis (PDD), using 5-aminolevulinic acid (ALA), is one of these tools. In this method, ALA is administered orally, and a high concentration of ALA in cancer cells, results in their red appearance in blue light URS [24]. The most common side effect of this method is hypotension, which is mild in nature [25]. PDD has shown improvements in detecting CIS but its application in UTUC diagnosis is limited since it requires dedicated ureteroscopes, and its highest quality is achieved when the tissue being observed is positioned at a perpendicular angle to the ureteroscope, while in URS, the mucosa is mainly parallel to the probe [25,26].

Narrow band imaging (NBI) is a technique that uses two narrow bands of white light, which are taken up by hemoglobin in the blood vessels, and theoretically, makes identification of tumors easier. Only two papers have been published on its application at UTUC [27,28]. In 2011, Traxer et al. published a series of 27 patients that were simultaneously inspected by both NBI and white light. NBI improved visualization and tumor detection by 22.7% [27]. In 2018, Lordache et al. used NBI on 87 patients and concluded that NBI improves the detection rate of pTa and CIS [28].

Optical coherence tomography (OCT) is a tool that is used in ophthalmology to visualize retinal layers. It is based on light emission, reflection and scattering. It has been used with flexible URS to assess the depth and penetration of the tumor. Its main drawback is that it is limited to approximately 2 mm of depth. Although it discriminates between invasive and non-invasive tumors, it is not helpful in cases of more advanced disease. It can also assess tumor grade by measuring the decrease in light intensity [29]. When compared to biopsy, it has shown superior results in terms of staging and grading of the tumors [30].

Confocal laser endomicroscopy (CLE) is a novel tool that is implemented in UTUC diagnosis. After introducing fluorescein to the tissue (either intravenously or topically), a probe is introduced to the urothelium through the URS. Excited fluorescein emits light that is absorbed through a pinhole, which ultimately gives a picture almost identical to histology. The main difference is that fluorescein cannot cross cell membranes, so it does not show nuclear features. It is also applicable to biopsied tissues [31]. Currently, there are three small patient series published on in-vivo use to diagnose UTUC [32,33,34]. Taken all together, CLE can correctly assess low-grade UTUC in a high percentage of patients but is less accurate in high-grade disease. Despite excellent results, more studies with bigger patient populations need to be executed.

Although great results have been documented with novel diagnostic tools, their place in the diagnostic spectrum of UTUC is not well established yet.

Since ureteroscopic biopsy can be inaccurate in assessing tumor stage, and is associated with an increased risk of post-RNU IVR, the EAU guideline favors performing URS without biopsy [6]. In terms of biopsy devices, the largest specimens are obtained using BIGopsy backloading biopsy forceps in flat and sessile lesions, and by using Nitinol basket biopsy in papillary tumors [35,36]. However, one study questioned the BIGopsy forceps utility, considering its huge size, backloading requirement, and blocking the field of view [37]. The standard 3F forceps (Piranha) is considered inferior to both of them [35,36].

Novel techniques are proposed to increase the quality of specimens obtained through biopsy. Cryobiopsy involves using a cryoprobe to create an ice ball around the tissue of interest through sudden decompression of carbon dioxide. This technique allows for effective biopsy as the ice ball adheres more strongly to the probe than to the surrounding tissue. Compared to standard biopsy tools, the use of cryoprobes have been found to produce larger and higher-quality biopsies, more representative of the original tissue structure. The implementation of this technique in clinical settings has the potential to yield promising results, as shown by an ex-vivo study. However, it requires to be confirmed by rigorous in-vivo studies [38].

In the “form tackle” technique, a cold cup biopsy forcep is introduced through the ureteroscope. It is opened and pressed at the base of the lesion to include the submucosal tissue. The forceps are advanced 3–10 mm, and then pulled. The preliminary data based on fourteen patients who went through this procedure indicated that this method provides larger specimens [39].

Obtaining a biopsy without URS has been investigated as well. Percutaneous core-needle biopsy (PCNB) was shown to be feasible, accurate, and safe for UTUC diagnosis [40]. Joseph et al. reported the results of PCNB, guided by computed tomography (CT) or ultrasonography (US), prior to RNU. PCNB provided tumor grade in 69% of the cases, and of these, 89.7% were concordant with the final pathology. No tract seeding was identified during the 28 month follow-up [41].

### 3.3. Risk Stratification

Risk stratification aims to guide therapeutic decisions regarding the type of surgery (radical vs. kidney sparing) and peri-operative systemic therapy (neo- and adjuvant chemotherapy). Patient- and tumor-related prognosticators along with various biomarkers are used for this purpose.

## 4. Patient-Related Prognosticators

### 4.1. Age and Sex

A meta-analysis revealed a weak significant association between advanced age and overall survival (OS) (HR: 1.05), progression-free survival (PFS) (HR: 1.01), and cancer-specific survival (CSS) (HR: 1.02) [42]. Another meta-analysis on post-operative nomograms revealed a significant negative predictive value of age for CSS [1]. Although several studies have shown the association between age and survival indices [43,44], no association was found after adjustment for performance status (PS) [45]. However, age is found to be a predictor of muscle-invasive disease [46,47,48,49].

Unlike bladder cancer, UTUC prognosis is not associated with gender [50].

### 4.2. Ethnicity

One study indicates differences in clinicopathological features and OS between United States and Chinese patients, with US patients having a worse OS (*p* = 0.049) [51]. Another study suggests worse cancer-specific mortality (CSM) in Asian ethnicity compared to Caucasians (HR: 1.29, *p* < 0.01), after PS-matching. This study did not find any difference in tumor grade or T-stage between studied ethnicities (Asian, Caucasian, Hispanic, and African American) [52]. A shorter survival is suggested for African Americans, without a clear explanation of whether it is related to access to care or biological differences [53].

### 4.3. Tobacco Consumption

While smoking ≥20 cigarettes per day for ≥20 years, increases the chance of advanced disease stage, disease recurrence, IVR after RNU, and mortality; its detrimental effects are mitigated after 10 years of cessation [54,55]. A meta-analysis with 2259 patients showed a strong association between smoking and disease recurrence (HR: 1.57, 95% CI = 1.19–1.95), and CSM (HR: 1.53, 95% CI: 1.13–1.92) [56].

### 4.4. Surgical Delay

Waiting more than 120 days between diagnosis and definitive surgery was associated with lower OS in 3581 UTUC patients who underwent RNU [57]. Sundi et al. found no significant difference in survival outcomes in their group of 186 patients who were divided into early (<3 months) and late (≥3 months) surgery groups [58]. Considering studies on surgical waiting time, the EAU recommendation remains to perform definitive surgery within the first 12 weeks of diagnosis [6].

### 4.5. Other Factors

A meta-analysis of 81,814 patients with solid tumors indicates a prevalence of 35.3% for sarcopenia [59]. A recent cohort of 142 patients found that sarcopenia is a common finding in UTUC (prevalence: 37.3%). This suggests that its prevalence in UTUC does not differ from other solid tumors. Moreover, the study found sarcopenia as a comorbidity-independent predictive factor for OS (HR: 1.77; 95% CI: 1.02–3.07; *p* = 0.042) and CSS (HR, 2.17; 95% CI 1.18–3.99; *p* = 0.012) in UTUC patients following RNU. The authors also suggested that a high visceral adipose tissue index measured on a CT-scan at the height of third lumbar vertebra is associated with better outcomes after RNU. However, this finding was not statistically significant [60].

Pre-operative nutritional status is a significant determinant of survival outcomes. The preoperative prognostic nutritional index (PNI) is calculated by the following formula: PNI = 10 × serum albumin concentration (g/dL) + 0.005 × lymphocyte counts (number/mm^3^). Low PNI is associated with poorer OS and PFS. Nutritional support and possible postponement of surgery until better general status is achieved is suggested in patients with low PNI [61,62]. Albumin level is used to calculate the HALP (hemoglobin, albumin, lymphocyte, and platelets) score as well. Gao et al. divided 533 UTUC patients who underwent RNU into low- and high-HALP groups. Lower HALP score was associated with poorer OS (HR = 1.54, 95% CI, 1.14–2.01, *p* = 0.006) and PFS (HR = 1.44, 95% CI, 1.07–1.93, *p* = 0.020) [63]. A negative correlation of low pre-operative Albumin (<39.8 g/L) with OS, PFS, and CSS was reported by Zhao et al. [64]. 

Zhao et al. also combined decreased albumin with elevated neutrophil-to-lymphocyte ratio (NLR) and divided patients into three groups of having none, either one, or both of these factors. The 5-year PFS rate dropped from 77.8% to 52.6% to 32.3%, the 5-year CSS rate dropped from 97.7% to 71.4% to 32.9%, the 5-year OS rate dropped from 92.7% to 70.4% to 29.2%, in respective groups (all *p* < 0.0001) [64]. Increased pre-operative NLR was shown to be predictive of poor OS (HR: 1.72, 95% CI: 1.45–2.05), PFS (HR: 1.68, 95% CI: 1.44–1.96), and CSS (HR: 1.64, 95% CI: 1.39–1.93), in a meta-analysis on 11,538 patients from 32 studies [65].

Elevated pre-operative fibrinogen is another marker associated with worse OS (HR: 2.09; *p* < 0.001), RFS (HR: 2.09; *p* < 0.001), and CSS (hazard ratio [HR]: 2.33; *p* < 0.001) [66]. Egger et al. combined elevated fibrinogen with high C-reactive protein and showed that concomitant elevation of both factors is associated with adverse histological characteristics. The score based on these factors was predictive of worse CSS in multivariate analysis and of OS in univariate analysis [67].

Traditional habits such as the use of herbs and plant food supplements, especially common in eastern societies, are known as an important risk factor contributing to the disproportionately high incidence of UTUC in Taiwan. Aristolochic acid (AA)-containing Chinese herbal preparations was banned in 2003. However, a recent study showed an increasing trend in the incidence of UTUC in Taiwan that may be attributed to the consumption of unknown sources of AA. This highlights the importance of vigorous surveillance of phytotherapy and herbal products, as they are gaining popularity in the modern world [68]. AA exposure results in aristolactam (AL)-DNA adduct formation. AL-DNA adducts are poorly repaired, hence remaining in target organs for years. These adducts can be used as biomarkers of AA exposure, and are found in a high proportion of Taiwanese UTUC patients [69,70,71]. The AL-DNA adducts result in A:T to T:A transversion, as a mutational signature [72]. AA-related UTUC was shown to be associated with higher grade, and stage of the tumor. However, it was not associated with increased IVR [73].

## 5. Tumor-Related Prognosticators

### 5.1. Tumor Stage and Grade

Tumor stage and grade are two well-established prognostic factors of UTUC. High-grade is associated with advanced stage, loco-regional and distant recurrence, and non-organ-confined (NOC) disease [74]. It is also associated with worse RFS (HR: 2.0, *p* < 0.001) and CSS (HR: 1.7, *p* = 0.001) [75]. In a Dutch series of 13,314 UTUC patients, the 5-year relative survival rates for superficial, organ-confined, and NOC disease were 85.7%, 69.6%, and 43.6%, respectively [76]. Both uni- and multi-variate analyses on 374 patients with primary localized UTUC revealed a higher risk of IVR in patients with higher-grade tumors (Relative risk (RR): 3.776, *p* < 0.0001) [77].

Katayama et al. argued that factors used in current risk stratification models (including that of EAU and National Comprehensive Cancer Network (NCCN)) other than clinical tumor stage and grade, do not add significant predictive value in clinically low-stage low-grade tumors. However, they limit the adoption of KSS. They proposed a model solely based on grade and stage (GS model), from the data of URS biopsy and imaging, that yielded comparable accuracy to that of EAU and NCCN and considered a higher portion of patients as candidates for KSS [78].

### 5.2. Tumor Presentation, Location, Multifocality, and Size

A recent study assessed the association between flank pain (FP), gross hematuria (GH), and survival outcomes in UTUC patients who underwent RNU. Unlike GH, the presence of FP was associated with worse 5-year OS (47.2% vs. 81.2% (FP+ vs. FP−), *p* = 0.001) and CSS (50.2% vs. 83.9%, *p* < 0.001). Multivariate analysis revealed FP, multifocality, and pathological stage as independent prognostic factors for OS and CSS. On subgroup analysis, the patients in group ‘FP without GH’ had the worst oncological outcomes. Patients with FP had a 2.95 times higher hazard ratio for cancer-specific death (CSD), compared to those without FP [79]. In a cohort of 2662 patients, 80% presented with hematuria (microscopic or gross), while only 15% presented with symptomatic hydronephrosis (i.e., hydronephrosis and FP). Hematuria was associated with less hydronephrosis, renal pelvic tumors, and early pathological tumor stage. Meanwhile, symptomatic hydronephrosis was associated with ureteral tumors and advanced pathological stage. On multivariate analysis, hematuria was linked with better OS (HR 0.789, 95% CI 0.661–0.942) and CSS (HR 0.772, 95% CI 0.607–0.980), while symptomatic hydronephrosis was a predictor of poorer OS (HR 1.387, 95% CI 1.142–1.683) and CSS (HR 1.587, 95% CI 1.229–2.050) [80]. One possible explanation is that the obstruction caused by ureteral tumors, results in asymptomatic hydronephrosis and the absence of hematuria, leading to tumor upstaging. Pre-operative hydronephrosis, irrespective of pain, was shown to be associated with advanced pathological and poor survival outcomes [81,82].

A meta-analysis of 14,895 patients indicated a pooled hazard ratio of 1.52 (*p* < 0.001) and 1.39 (*p* = 0.004) for CSS and OS in patients with ureteral involvement [83]. Another study on 11,922 patients revealed lower median OS for patients with ureteral involvement compared to pelvicalyceal tumors (66.8 vs. 71.1 months; *p* = 0.01) [84]. Moreover, the microenvironment of tumors arising from either of the two locations differs in immunological profile [85]. Miyake et al. proposed a site-specific risk stratification model for ureteral and renal pelvis tumors to predict extraurinary tract recurrence (EUTR), CSD, and IVR after RNU. They found that the site-specific models yielded a higher discriminative accuracy, compared to the overall UTUC risk model for all three end-points [86]. Multifocal tumors are associated with worse CSS [79]. In the study of Miyake et al., multifocality was a common risk factor in both ureteral and pelvicalyceal models [86].

A meta-analysis of 35 studies and 32,292 patients found that an increase in tumor size is significantly associated with decreased OS, CSS, RFS, and IVR rates (HR: 1.42, 95% CI: 1.28–1.58, *p* < 0.00001; HR: 1.66, 95% CI: 1.47–1. 88, *p* < 0.00001; HR: 1.25, 95% CI: 1.13–1.38, *p* < 0.0001; HR: 1.12, 95% CI: 1.04–1.20, *p* = 0.003; respectively). The authors attributed the positive associations between tumor size and poor outcomes in UTUC to several theories on the biological mechanisms. Large tumor size correlates with aggressive tumor behavior including advanced-stage, lymphovascular invasion (LVI), lymph node metastasis, tumor necrosis, and tumor multifocality. Larger tumors are more susceptible to LVI, a prerequisite for lymph node metastases, which significantly increases the risk of disease recurrence, and cancer-specific and overall mortality even after RNU. Moreover, extensive tumor necrosis (>10% of tumor area) has been reported to be associated with metastasis- and cancer-related deaths. Lastly, patients with larger tumor sizes are more likely to involve both the ureter and the renal pelvis, making open RNU necessary, and putting the patient at risk of poorer surgical outcomes [87,88,89].

Although, in one study, the tumor size with the cutoff of >2 cm was shown to be associated with muscle invasion (OR 2.38, 95% CI 1.70–3.32; *p* < 0.001) [90], several studies did not show tumor size as a predictive factor for muscle-invasive disease [47,91,92].

### 5.3. Lymphovascular Invasion

A recent meta-analysis of 58 studies comprising 29829 UTUC patients who underwent RNU, showed that LVI was present in 26.2% of patients, which makes it a common histopathologic finding in RNU specimens. LVI was found to be a significant predictor of disease recurrence (pooled HR: 1.43, 95% CI: 1.31–1.55, *p* = 0.000; I(2) = 76.3%), CSS (pooled HR: 1.53, 95% CI: 1.41–1.66, *p* = 0.000; I(2) = 72.3%), and OSS (HR: 1.56, 95% CI 1.45–1.69, *p* = 0.000; I(2) = 62.9%) [93]. Another study indicated an association between LVI and OS (HR 4.980 CI 95%1.763–14.064, *p* = 0.002), and PFS (HR 2.687 CI 95%1.172–6.163, *p* = 0.020) [94].

The systemic immune inflammation index (SII) is calculated by multiplying NLR by platelet count. Positive LVI was found to be significantly associated with advanced tumor stage, high tumor grade, tumor necrosis, lymph node metastasis, and high SII levels. The co-existence of positive LVI and high-level SII was further found to be a significant predictor of poorer OS, CSS, and PFS (with hazards ratios and 95% confidence intervals of 3.918 [2.168–7.078], 5.623 [2.679–11.801], and 3.377 [2.138–5.334], respectively). However, on further analysis, the effect of co-occurrence of LVI and SII on survival outcomes was significant only in NOC disease [95]. LVI is also predicted by increased NLR (HR = 1.29, 95% CI = 1.17–1.43). Increased NLR also predicts higher tumor stage and grade (HR: 1.25, 95% CI = 1.12–1.39; and HR: 1.07, 95% CI = 1.01–1.14; respectively) [65]. LVI occurs during the early metastatic phase by invasion of tumor cells to the lymphatic/vascular channels. It represents the dynamic state of the disease. High SII is an indicator of pro-tumor inflammatory response and a weak anti-tumor immune state (as implied by high neutrophil and platelet and low lymphocyte count) [95].

### 5.4. Surgical Margins

Positive surgical margin, following RNU, is associated with a higher chance of metastases (5-year metastasis-free survival (MFS) of 51.6% vs. 79.3%) [96]. A positive margin was found to be associated with lower MFS [97]. Pooled analysis of 37984 patients from eight comparative trials revealed that robot-assisted RNU was associated with significantly lower positive surgical margins, compared to open RNU (OR 0.33, 95% CI 0.12, 0.92; *p* = 0.03) [98].

### 5.5. Lymph Node Status

Poor overall survival comes with nodal metastasis [99]. A study on 306 node-positive patients indicated that the number of removed or positive lymph nodes was not associated with survival indices. Meanwhile, positive lymph node density (best cutoff = 27%) was associated with lower OS and CSS (HR: 1.62, *p* = 0.036, and HR: 1.75, *p* = 0.014, respectively). The 5-year OS rate for patients with density < 27% was 18.7%, compared to 34.2% for those with density ≥ 27% (*p* < 0.05) [100].

### 5.6. Mutational Landscape

UTUC has distinct genetic characteristics. Various mutations are common in UTUC including FGFR3, KMT2D, KMT2a, TP53, and MDM2. A recent robust study proposed a mutational classification flow chart for UTUC, composed of 5 subgroups: the hyper-, TP53/MDM2-, RAS-, FGFR3-, and triple-negative-mutated subtypes. These subgroups differ in prognosis. The triple-negative-subtype shares a similar prognosis to the TP53/MDM2-mutated subtype, which exhibits the most aggressive clinical course. On the contrary, low-grade histology and higher survival rates are seen in the FGFR3-mutated subtype. The RAS-mutated subtype is characterized by high-grade tumors and squamous cell differentiation [101,102].

By performing unsupervised hierarchical clustering, Su et al. identified two DNA methylation-based epi-clusters. Frequent hyper-methylation was witnessed in the EpiC-C1 cluster, which was more frequently associated with muscle-invasive UTUC, and shorter OS. The Epic-C2 was hypo-methylated, enriched in FGFR3 mutation, and associated with non-muscle invasive disease [103].

### 5.7. Other Factors

A meta-analysis of 14,368 patients revealed the significant association of sessile tumor architecture with disease recurrence and CSM (pooled HR: 1.454, and 1.416, respectively) [104]. In a study on 811 patients, sessile architecture was an independent predictor of muscle-invasive disease at RNU (*p* < 0.0001) [47]. In another study on 1214 patients who underwent RNU, sessile architecture was significantly associated with muscle-invasive or node-positive disease (OR: 2.31, 95% CI 1.58–3.36, *p* < 0.001) [49]. Papillary configuration was associated with a higher risk of IVR (RR: 3.244 *p* < 0.0001) [77].

Concomitant carcinoma in situ is associated with worse CSS and RFS (HR: 1.25; *p* = 0.004, and HR: 1.24; *p* = 0.006, respectively) [105]. Urothelial bladder cancer occurring at the same time (synchronous) or a different time (metachronous) with UTUC is a predictor of worse PFS (HR: 3.326 CI 95% 1.474–7.503, *p* = 0.004), but not OS [94].

Histological variants of UTUC are associated with the presence of adverse pathological features including higher stage and grade, tumor necrosis, positive surgical margins, and lymph node invasion. The micropapillary variant is associated with worse recurrence, and the sarcomatoid variant is linked to worse CSM. However, variant histology was not associated with survival outcomes in multivariate analyses [106,107].

A recent study assessed the tumor-stroma ratio according to histologic sections. It indicated an association between high-stroma tumors, poorer survival outcomes, and inferior responsiveness to chemotherapy. In addition, a correlation was shown between high-stroma tumors and immuno-evasive microenvironment with exhausted CD8^+^ T-cells [107].

## 6. Pre-Operative Predictive Tools

Due to the imperfection of imaging, endoscopy, and biopsy, it is still difficult to achieve precise preoperative characterization of UTUC in terms of grading, staging, and prognosis. Mori et al. indicated a huge discordance between pre-operative clinical and post-operative pathological staging and grading. URS biopsy underestimated the stage in 59.5% of patients. Final pathology of 89.6% of patients with clinical ≤ cT1 disease, indicated muscle-invasion. Concordance between clinical and pathological grading occurred in 54.2% of patients [48]. Despite these, CT urography and URS biopsy are still the main sources of pre-operative information, and several predictive models are designed by employing data obtained from these modalities and combining them with other prognosticators. Table 2 summarizes the features of 10 multivariable models that predict muscle-invasive/NOC disease.

Brien et al. used the data of 172 patients from five centers in the US and developed a pre-operative risk group stratification combining ureteroscopic biopsy grade, hydronephrosis, and urine cytology. Their model yielded 100% negative predictive value (NPV), when all three factors were negative, and positive predictive value (PPV) of 73% and 89% for NOC and muscle-invasive disease, respectively [108]. Using data from 274 patients from a single center in the US, Favaretto et al. proposed a risk group stratification model composed of ureteroscopic grade, tumor location, hydronephrosis, and invasion on imaging. Their model had an accuracy of 70% and 71% NOC and muscle-invasive disease, respectively [91]. These two models were not validated by the authors.

Margulis et al. studied 659 patients across 13 centers, mostly in the US and Europe, and developed an internally validated model for NOC prediction with 76.6% accuracy. Their nomogram comprised ureteroscopic tumor architecture, grade and location [109]. Chen et al. developed a nomogram using data from 693 Chinese patients from a single center. Gender, tumor architecture, multifocality, location, grade, and hydronephrosis were predictive factors of this internally validated model with 79% accuracy for NOC and muscle-invasive disease prediction [110]. Singla et al. compared predictive factors of NOC in UTUC patients from the US and China. They indicated that in the US cohort, clinical T3 stage and high-grade pathology on ureteroscopic biopsy were significant NOC disease predictors. Significant predictors of NOC in the Chinese cohort were male gender, tumor location and size on imaging, NLR, and pre-operative estimated glomerular filtration rate (eGFR). They further applied Margulis et al. and Chen et al. models to their study cohorts and found that the Western model (i.e., Margulis et al.) has an accuracy of 75% and 67% in the US and Chinese cohorts, respectively. The Chinese model (i.e., Chen et al.), was 76.3% and 82.8% accurate for US and Chinese populations, respectively [112]. Their results, somehow externally validated these models, and proposed that population-based differences should be considered during the clinical application of predictive models.

Petros et al. developed a nomogram for NOC disease prediction, using data from 566 patients from three centers in the US. The predictive factors used are ureteroscopic grade, tumor architecture, clinical stage, and pre-operative serum hemoglobin. An accuracy of 82% was achieved in the development cohort. Internal and external validation was performed and the model showed 77% accuracy in the test cohort. They further suggested an easily-remembered cut-off point ≥ 0.49 for high-risk disease on their nomogram [92]. Yoshida et al. used two independent Japanese databases to develop and validate a nomogram for NOC disease prediction. Their nomogram composed of NLR, chronic kidney disease (CKD), tumor location, hydronephrosis, and local invasion on imaging, achieved 77% accuracy [111]. URS data are not implemented in the Yoshida et al. nomogram, hence its applicability in patients whose UTUC is detected in imaging upon initial evaluation. These two models can be effectively applied to select patients for pre-operative systemic therapy.

Foerster et al. performed an international multi-institutional study and analyzed data of 1214 patients from 21 centers across North America, Europe, and Eastern Asia. Multivariate logistic regression analysis revealed invasion on imaging, biopsy cT1+ staging, sessile architecture, high-grade biopsy, hydronephrosis, tumor size, and age (OR: 5.10, 3.23, 2.31, 1.81, 1.37, 1.09, 1.02, respectively), were significantly associated with ≥pT2/N+ disease. In addition to these factors, they employed four other factors (previous radical cystectomy, multifocality, cytology, and sex) to develop an internally validated nomogram with a resultant bias-corrected accuracy of 75%. The additional clinical net reduction of 4 per 100 patients over the EAU model, is a superiority. This means using this model in a probability threshold of 20–40%, prevents up to 4 additional patients per 100 from unnecessary RNU, meaning they can benefit from kidney-sparing surgeries. They emphasized the robust role of biopsy staging and tumor architecture in NOC disease prediction, as two factors not used in the EAU risk stratification model [49]. An odds ratio (OR) of 9 was reported for the clinical T stage of 1+ in the prediction of muscle-invasive disease [113]. Foerster et al. indicated that Applying tumor architecture is the advantage of the NCCN model over the EAU model [49].

In their 2022 study, Marcq et al. strived to find predictors of muscle-invasive disease. Non–organ-confined disease on preoperative imaging, sessile architecture, hydronephrosis, high-grade cytology or biopsy, and higher age at diagnosis were found significant in the multivariable analysis of data from 1214 patients from 21 centers. They proposed a new trichotomous classification in contrast to the dichotomous risk categories of EAU guidelines, categorizing UTUC patients as low- intermediate- and high-risk. Due to limitations imposed by tumor size on endoscopic management, Marcq et al. kept this factor along with other significant predictors found on multivariate analysis, as indicators of high-risk disease. Previous radical cystectomy and tumor multifocality are used to divide non-high-risk patients into low and intermediate groups. In comparison to the low-risk group, the odds ratios for muscle invasion were 5.5 (95% CI: 1.3–24.0; *p* = 0.023) and 12.7 (95% CI: 3.0–54.5; *p* = 0.0006) for intermediate- and high-risk groups, respectively. Their model’s area under the curve was 77% [47].

Venkat et al. identified 6143 patients from the National Cancer Database, who underwent extirpative surgery and lymph node dissection. LVI, ureteroscopic grade, positive clinical lymph node status, tumor size, and patient age were predictors of muscle-invasive disease. Node-positive disease predictors were positive clinical lymph node status, LVI, ureteroscopic grade, and tumor size. They developed two nomograms for the prediction of muscle-invasive disease, particularly to decide on administering neo-adjuvant systemic therapy, and node-positive disease, to guide the extent of lymph-node dissection. One advantage of their nomograms is that they offer and unknown/indeterminate option for LVI, tumor grade, and clinical lymph node status. This will allow the physician to estimate the probability of muscle-invasive or lymph-node-positive disease despite the lack of data on those factors. Their internally validated nomograms have an accuracy of 80%, and 87.8% for muscle-invasive, and positive-node disease prediction, respectively [46].

Besides nomograms for the prediction of muscle-invasive or NOC disease, studies were carried out to develop nomograms predicting pathologic grade and renal insufficiency following RNU. Ma et al. indicated that ureteroscopic biopsy high-grade, positive urinary cytology, sessile architecture, and age (ORs: 10.85, 6.87, 3.86, and 1.03, respectively; all *p*-values < 0.05) were pre-operative predictors of pathological high-grade following RNU. The corresponding nomogram, which was developed based on data from 245 patients from one center in China, achieved an Area under the ROC Curve (AUC) of 78%. This nomogram helps reduce the likelihood of undergrading by URS biopsy [114]. 

A study by Fang et al. on 606 Chinese patients showed that older age, tumors with smaller size, or located in the renal pelvis, lower preoperative eGFR, and the absence of hydronephrosis or multifocality were significant predictors of decreased renal function after RNU. They developed two nomograms for predicting ineligibility for full-dose and reduced-dose adjuvant chemotherapy with accuracies of 75.7% and 83.6%, respectively. Furthermore, they showed postoperative renal function did not have any correlation with patients’ survival [115]. Analyzing data from 226 patients from 17 institutions worldwide, Wu et al. developed a nomogram incorporating age, pre-operative eGFR, hydroureteronephrosis, and body mass index (BMI) to predict renal function <50 mL/min/1.73 m^2^ following RNU. They performed external validation on an additional 135 patients, which confirmed the 77% discrimination ability of the nomogram [116].

## 7. Post-Operative Predictive Tools

Post-operative risk stratification helps physicians decide on administering adjuvant chemotherapy and plan the follow-up strategy. Various post-operative nomograms have been developed to predict oncological outcomes in UTUC patients. A recent systematic review and meta-analysis comprehensively sums up these nomograms up to December 2021 [1]. Twenty-six nomograms were identified, only four of which were externally validated. It was not possible for authors to pool the concordance index (c-index) of each nomogram separately, so they categorized nomograms into four groups and calculated the overall performance of each group. Nomograms predicting OS, CSS, RFS, IVR after surgery, and CSS at the time of IVR were respectively assigned to groups A through E. The c-index for nomograms in groups A, C, and D (Predicting OS, RFS, and IVR following surgery) was >0.6, while this value was >0.7 for group B (predicting CSS). The most reliable negative predictors of OS, and RFS, were pathological tumor stage (pT) 3 or higher, and LVI, respectively. CSS was most reliably predicted by ≥pT2, age, and LVI [1].

This review emphasizes the absence of external validation studies and data limitations regarding clinical utility. It encourages the design and conduct of studies to address these issues, which would yield in the clinical applicability of post-operative nomograms. The authors further provide reference tools to help physicians implement appropriate post-operative nomograms according to their individual needs [1].

Tian et al. developed a nomogram for the prediction of OS in UTUC patients receiving chemotherapy. They extracted data from 1195 from the SEER database and found age, TNM stage, marital status, and surgical methods of the primary site, as significant predictors of OS. The AUC values of 78.9%, 77.2%, and 76.3% show discrimination accuracy of their nomogram for 1-, 3-, and 5-year OS in the development cohort. Their internally validated nomogram showed superior accuracy compared to the American Joint Committee on Cancer (AJCC)-TNM staging system [44].

Recent EAU guideline on UTUC recommends adjuvant platinum-based chemotherapy following RNU to patients with pathologic muscle-invasive or node-positive disease. It also suggests discussing adjuvant nivolumab with patients with NOC disease following RNU, who did not receive neo-adjuvant chemotherapy and refused platinum-based adjuvant chemotherapy, or are not fit for it [6]. Proper therapeutic decision-making and patient counseling require judicious application of post-operative nomograms, for which the mentioned studies would be of greatest help.

## 8. Future Directions

The current EAU risk-stratification tool for non-metastatic UTUC dichotomizes patients into low- and high-risk groups. If the tumor is unifocal, <2 cm, low-grade on URS biopsy, negative for high-grade cytology, and with no invasion on CT imaging, it is considered low-risk; hence a candidate for KSS. Otherwise, it should undergo RNU. 

Overall, with advances in minimally-invasive management and evidence of acceptable oncological outcomes in well-selected patients who underwent conservative surgeries, a revision on the current stratification of non-metastatic UTUC seems a sage act. In this regard, considering proposed stratifications deviating from the classic dichotomizing stratification tool, similar to those of Marcq et al. [47], or Benamran et al. [117] would be helpful.

## 9. Conclusions

There are various diagnostic tools able to enhance the current techniques used for diagnosing UTUC. Novel proposed pre- and post-operative nomograms can guide the path of management with acceptable accuracy. These new diagnostic and risk stratification tools require further validation by robust prospective studies conducted on an international multi-institutional collaboration basis. Only then, these tools will be applicable to clinical practice, resulting in a greater number of patients benefiting from kidney-sparing procedures.

## Figures and Tables

**Table 1:** Summary of studies reporting the association between URS and IVR, and RFS.

Study	Patient Population/Study Duration	No. of Patients	Median Follow Up	Urinary Bladder Recurrence	Recurrence Free Survival	Median Time to Recurrence	Cancer-Specific Death	Comments
Liedberg F, et al. (2023) [8]	Sweden 2015–2019	1038 IDM+: 536 IDM−: 502	1.3 yrs	220 (21.2%) IDM+: 120 (22.38%) IDM−: 100 (19.20%)			IDM+: HR: 1.56 95% CI: (1.12–2.18)	IDM increases risk of IVR in ureteric tumor and not in the renal pelvis
Luo Z, et al. (2023) [9]	China 2009–2020	220 1-session URS: 22 (10%) 2-session URS: 112 (51%) No URS: 86 (39%)	41 mos	58 (26.4%) 1-session URS: 5 (22.7%) 2-session URS: 36 (32.1%) No URS:17 (19.8%)				Delayed RNU following URS (2-session) could increase the IVR risk, but not immediate RNU after URS (1-session)
Anbarasan T, et al. (2023) [10]	UK 1998–2015	267		73 (27.3%)	5-yr RFS 64.7% URS + Bx: 49.9% URS−: 76.4%			Identical mutational changes in genes (TP53 and FGFR3) between primary UTUC and subsequent IVR.
Douglawi A, et al. (2022) [11]	USC-USA 2005–2019	143 URS+: 104 (73%) Access sheath+: 36 (25%) No URS: 39 (27%)	27 mos	36 (25%) URS+: 30.8% (Access sheath+: 11.5% Access sheath−: 39.7%) No URS: 7.7%		URS+: 9.0 mos No URS: 12.1 mos		URS increases IVR but using an access sheath may mitigate this effect
Ha JS, et al. (2022) [12]	R Korea 2016–2019	396 Rigid URS: 178 (45%) Flexible URS: 111 (28%) No URS: 107 (27%)	1 yr	99 (25%) Rigid URS: 41 Flexible URS: 37 No URS: 21				Rigid URS may not increase the risk of IVR, whereas flexible URS appears to be associated with a higher risk of IVR.
Sharma V. et al. (2021) [13]	USA 1995–2019	834 no URS: 210 (25.2%) Percutaneous Bx: 57 (6.6%) URS-Bx: 125 (15%) URS + BX: 442 (53%)	2 yrs	No URS: 15% Percutaneous Bx: 12.7% URS-Bx: 18.7% URS + BX: 21.9%				URS + Bx but not percutaneous Bx or URS-Bx increases IVR risk
İzol V et al. (2021) [14]	Turkey 2005–2019	194 URS+: 95 (49% URS−: 99 (51%)	39.17 mos	54 (27.8%) URS+: 38.9% URS−: 17.2%	URS+: 60 mos URS−: 111 mos	10 mos		URS was associated with poor recurrence free survival
Shsm H, et al. (2021) [15]	UK 2012–2019	69 URS+: 49 (71%) URS−: 20 (29%)	48.5 mos	URS+: 28.3% URS−: 5.9%				Diagnostic URS delays definitive treatment and is associated with higher IVR
Chung Y, et al. (2020) [16]	Korea 2003–2018	453 URS+: 226 (49.9%) URS−: 227 (50.1%)	15 mos	URS+: 99 (43.8%) URS−: 61 (26.9%)	5-yr URS+: 56.2% URS−: 73.1%			Preoperative URS increases IVR. It is better not to perform URS before surgery
Baboudjian M, et al. (2020) [17]	France 2005–2017	93 URS+: 70 No URS: 23	35 mos	47 (50%) URS+: 41 (87%)		URS+: 226 days No URS: 427 days		High IVR rate after URS
Lee HY, et al. (2018) [18]	Taiwan 1990–2013	502 URS + Bx: 206, 41% No URS: 296, 59%	6.4 yrs	138 (27.5%) URS+ Bx did not increase IVR (*p* = 0.609)	URS+ = no URS (*p* = 0.829)			URS + Bx is not associated with higher risk of IVR
Lee HY, et al. (2018) [18]	Taiwan 1996–2013	5713 URS+: 3079 No URS: 2634		No URS: 392 (14.88%) URS + Bx: 515 (16.73%)	URS + Bx = no URS *p* = 0.442 in low grade *p* = 0.292 in high grade			URS + Bx do not increase IVR irrespective of the tumor location
Sankin A, et al. (2016) [19]	New York, USA 1994–2012	201 URS+: 144 (72%) URS−: 57 (28%)	5.4 yrs	89 URS+: HR 2.58; 95% CI 1.47, 4.54	3-yr RFS URS+: 42% URS−:71%			URS increases the risk for IVR but does not have an effect on disease progression or survival
Liu P, et al. (2016) [20]	Beijing, China 2000–2011	664 URS+: 81 No URS: 583	48 mos	223 (33.6%)	2-yr RFS URS+: 71.4% No URS: 79.3%	17 months		URS is independently associated with IVR
Sung HH, et al. (2015) [21]	Korea 1994–2013	630 URS+: 282 (44.7%) No URS: 348 (55.3%)	34.3 mos	268 (42.5%)	5-yr RFS URS+: 42.6 ± 8.0% No URS: 63.6 ± 6.9%			URS increases IVR but URS with manipulation does not have an effect IVR
Ishikawa S, et al. (2010) [22]	Japan 1990–2005	208 URS+: 55 (26.5) No URS: 153 (73.5%)	44 mos	86 (41.3%)	2-yr RFS URS+: 60% No URS: 58.7%			Diagnostic URS does not have an effect on IVR or cancer specific survival

IDM: Invasive Diagnostic Modalities, including all invasive workup tools such as antegrade/retrograde uretero-pyelography and/or selective urine cytology/barbotage, and URS with or without concomitant biopsy.

**Table 2:** Pre-operative predictive tools for muscle-invasive, NOC, or node positive UTUC.

First Author	Year	Prediction Form	Number of Patients	Prognosticators	Prediction of	Accuracy	Validation
Brien [108]	2010	Risk group stratification	172	Hydronephrosis, biopsy grade and urinary cytology	NOC UTUC Muscle Invasive	PPV 73 NPV 100 PPV 89 NPV 100	
Margulis [109]	2010	Nomogram	659	Tumor architecture, tumor grade and tumor location	NOC UTUC	76.6	Internal
Favaretto [91]	2012	Risk group stratification	274	Ureteroscopic grade, tumor location, Hydronephrosis and invasion on imaging	NOC Muscle Invasive	70 71	
Chen [110]	2013	Nomogram	693	Gender, architecture, multifocality, tumor location, grade and Hydronephrosis	NOC Muscle Invasive	79 79	Internal
Petros [92]	2018	Nomogram	566	Ureteroscopic grade, Architecture, Hemoglobin, Clinical stage	NOC UTUC	82 Development 77 Validation	Internal & External
Yoshida [111]	2020	Nomogram	1101	NLR, CKD, Tumor location, Hydronephrosis, Local invasion on imaging	NOC UTUC	77	Internal & External
Foerster [49]	2021	Nomogram	1214	Previous RC, architecture, multifocality, invasion on imaging, tumor size, Preoperative hydronephrosis, Cytology, Biopsy staging, biopsy grading, sex, age	≥pT2/N+	75 (bias corrected)	Internal
Marcq [47]	2022	Risk group stratification	1214	≥cT3, sessile architecture, hydronephrosis, High grade cytology, high grade biopsy, age at Dx	≥pT2	77	
Venkat [46]	2022	Nomogram	6143	Age, architecture, urine cytology, biopsy grade, LVI, Tumor size, cN	≥pT2	80	Internal
Venkat [46]	2022	Nomogram	6143	LVI, cN, Biopsy grade, tumor size	Positive Node	87.8	Internal

NOC: Non-organ confined; PPV: Positive predictive value; NPV: Negative predictive value; NLR: Neutrophil to lymphocyte ratio; CKD: Chronic kidney disease; RC: Radical cystectomy; Dx: Diagnosis; cN: clinical Node-positive.
